# Investigating the Effect of Consumers’ Knowledge on Their Acceptance of Functional Foods: A Systematic Review and Meta-Analysis

**DOI:** 10.3390/foods11081135

**Published:** 2022-04-14

**Authors:** Mathew T. Baker, Peng Lu, Jean A. Parrella, Holli R. Leggette

**Affiliations:** Department of Agricultural Leadership, Education and Communications, Texas A&M University, College Station, TX 77843, USA; peng.lu@ag.tamu.edu (P.L.); jean.parrella@ag.tamu.edu (J.A.P.); holli.leggette@ag.tamu.edu (H.R.L.)

**Keywords:** consumer acceptance, functional foods, knowledge

## Abstract

Inconsistent results published in previous studies make it difficult to determine the precise effect of consumer knowledge on their acceptance of functional foods. We conducted a systematic review and meta-analysis by identifying and collecting relevant literature from three databases. Of the 1050 studies reviewed, we included 40 in the systematic review and 18 in the meta-analysis. Based on the focus of each included study, we operationally defined knowledge as knowledge of the functional food concept, nutritional-related knowledge, and knowledge of specific functional products. Results from the systematic review indicate that most participants from the included studies had low knowledge, especially nutrition-related knowledge associated with consuming functional foods, and were generally not familiar with the concept of functional foods. Results from the meta-analysis generated a summary effect size (*r* = 0.14, 95% CI [0.05; 0.23]), measured by the correlation coefficient *r*, which indicates a small positive relationship exists between consumers’ level of knowledge and their acceptance of functional foods. Results from our study demonstrate the importance of increasing consumers’ functional foods knowledge to improve their acceptance of such products. Agricultural and health communicators, educators, and functional foods industry professionals should prioritize increasing consumers’ knowledge through their communications, marketing, and programmatic efforts.

## 1. Introduction

Consumers’ modern rapid lifestyle, characterized by improper nutrition intake and low physical activity, has contributed to the spread of chronic diseases (e.g., obesity, diabetes, heart disease, cancer) [1], which are leading causes of US deaths [2]. In fact, the Centers for Disease Control and Prevention estimated that more than half of US adults have at least one chronic disease. As a result, US medical expenditure resulting from chronic disease is the highest health expenditure worldwide, reaching about $3.7 trillion per year [2]. Previous evidence suggests that adequate nutritional intake could sustain normal physiological function to prevent diet-related chronic diseases [3]. Therefore, to improve the health and well-being of consumers, decrease health expenditures, reduce the risk of chronic diseases, and support human health, functional foods were developed [4,5].

Many definitions of functional food have been offered. Diplock et al. [6] proposed the widely accepted definition of functional foods—“Food can be regarded as functional if it is satisfactorily demonstrated to affect beneficially one or more target functions in the body, beyond adequate nutritional effects, in a way that is relevant to either improved stage of health and well-being and/or reduction of risk of disease” (p. 6). Moreover, functional foods include a large variety of products, produced using novel technologies (e.g., enrichment, fortification, enhancement) to remove compounds that negatively impact human health and replace them with functional ingredients that provide health benefits [7]. 

In recent years, consumers have become increasingly aware of chronic diseases and are willing to modify their diet to ensure adequate nutritional intake [8]. As a result, the functional food industry has tried to expand functional attributes to diversify functional products [9]. In general, however, consumers are hesitant to accept novel food technologies [10], including those involved in the development of functional foods. The nutritional characteristics of functional foods, coupled with the novel technologies used in their development process, pose challenges for consumers who lack familiarity and nutritional knowledge.

Consumers’ knowledge has a significant effect on their food choice behavior [11]. Topolska et al. [12] identified knowledge as the most important factor influencing consumer preferences and acceptance of functional foods. Previous studies found that consumers are willing to accept functional foods if they have enough knowledge to understand the health benefits of consuming such foods [13,14,15]. Specifically, to accept functional foods, consumers need to link their knowledge about such foods to relevant health benefits associated with their consumption [16]. Consumers who have higher levels of knowledge can better understand the health benefits of consuming functional foods and, therefore, are motivated to purchase such products because they can connect the health information presented on products’ labels with their knowledge [16,17,18]. 

Consumer research has been identified as one of the most important research areas to evaluate consumers’ acceptance of functional foods [1,19,20]. Many studies have investigated the relationship between consumers’ level of knowledge and acceptance of functional foods. However, the strength and direction of this relationship reported in previous studies vary greatly. For example, some studies found consumers’ knowledge positively influenced their acceptance of functional foods [17,18,21], and others found no significant relationship [8] or a negative relationship [22]. Consequently, these inconsistent results posed problems in determining the precise effect consumers’ knowledge has on their acceptance. 

To the best of our knowledge, no studies provide systematic evidence regarding the relationship between consumers’ knowledge and their acceptance of functional foods. Therefore, the purpose of our study was to provide a comprehensive overview of the peer-reviewed literature pertaining to consumers’ level of knowledge and determine the relationship between their level of knowledge and acceptance of functional foods. To achieve this purpose, three objectives guided the study: (1) Describe the included studies’ characteristics (e.g., country of origin, type of functional food, size and age range of the sample, type of knowledge, research design, outcome variable, key findings regarding consumers’ knowledge); (2) Qualitatively synthesize consumers’ level of knowledge as reported in the included studies; and (3) Identify the strength and direction of the relationship between consumers’ level of knowledge and their acceptance of functional foods.

## 2. Materials and Methods

### 2.1. Study Design

We conducted a systematic review (qualitative synthesis) and meta-analysis (quantitative synthesis) to achieve the study’s purpose. Using these methods, researchers seek to collect, combine, analyze, and present results from existing studies conducted on a specific topic using a predefined study protocol [23]. Together, a systematic review and meta-analysis can provide rigorous evidence and a comprehensive, unbiased overview of the body of knowledge on a specific topic [24]. Therefore, we chose to implement these methods because we sought to answer defined research questions through structured reviews of existing evidence [25]. We relied on the Preferred Reporting Items for Systematic Reviews and Meta-analysis (PRISMA) statement as a guide [26]. We also conducted a quality assessment of each included study to avoid potential reported biases or misleading results [23].

### 2.2. Database and Search Strategy

We systematically searched three databases—Web of Science (core collection), CAB abstracts (Ovid), and Food Science and Technology Abstracts (FSTA)—in July 2021. One author of this study and a subject librarian conducted the literature search. We selected relevant search terms related to functional food development technologies (e.g., enrichment, fortification, enhancement [7]) based on how they are described in previous research. We conducted a pilot test search to develop, test, and refine the search words. The combination of three sets of search strings included (functional food * or functional product * or enriched food * or enriched product * or fortified food * or fortified product * or enhanced food * or enhanced product *) and (accept * or behavior * or attitude * or perception * or pay or buy or purchase* or preference * or choice* or response *or reaction * or aware * or believe * or belief) and (knowledge). No limitation for the year of publication was applied (see the Supplementary Material Table S1 for the detailed search strings for the three databases). We also conducted a manual search through reference lists of included studies and similar published reviews to check for missing relevant studies. 

### 2.3. Eligibility Criteria

We had five eligibility criteria for study inclusion. We included studies (records) that quantitatively investigated the relationship between consumers’ knowledge and acceptance of functional foods. We excluded qualitative studies to avoid researcher biases and, additionally, because qualitative methodologies do not allow for the extraction of effect sizes. Each study needed to include consumer knowledge as a primary variable of interest. In addition, we included studies focused on modified or altered functional foods, excluding studies focused on nutraceutical foods or unmodified whole functional foods because consumers tend to be less trusting of modified functional foods. We further excluded studies that focused on functional foods processes (e.g., product development process, functional ingredients evaluation, packing methods). Finally, we included only studies written in English and published in peer-reviewed journals. 

**Figure 1:**
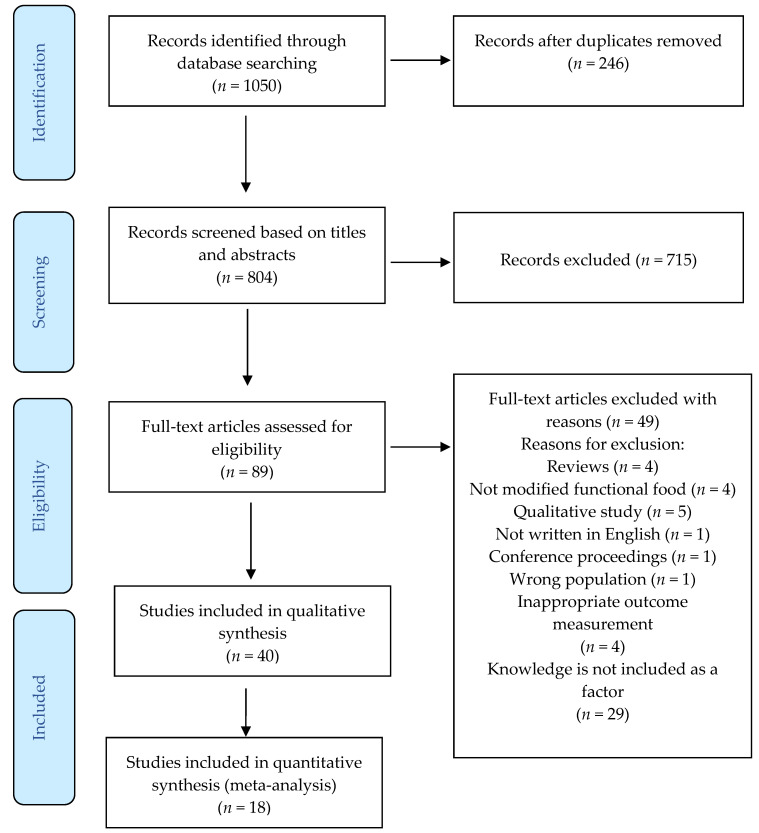
The PRISMA flow chart of study selection and screening.

The initial search of three databases generated 1050 studies, which were uploaded into Covidence, a citation management software for conducting systematic reviews. Two-hundred and forty-six (*n* = 246) duplicates were automatically removed from Covidence. Two authors individually conducted a title and abstract screening of the remaining 804 studies (*n* = 804) and excluded 715 (*n* = 715) because they did not qualify for our study based on the inclusion and exclusion criteria we established. Then, the same two authors conducted a full-text review of the remaining 89 studies (*n* = 89) and excluded an additional 49 (*n* = 49) that did not qualify. We addressed discrepancies through discussion. As a result, we included 40 studies (*n* = 40) in the systematic review, 18 (*n* = 18) of which provided sufficient quantitative data to be included in the meta-analysis. Figure 1 presents the flow chart of study selection and inclusion.

### 2.4. Data Extraction and Quality Assessment

The same two authors who conducted the title and abstract screenings and full-text reviews were also involved in data extraction, with one of us independently completing data extraction and the other thoroughly double-checking the work. We organized the extracted data into a Microsoft Excel spreadsheet and included authors’ names, the title of the article, publication year, country of origin, functional foods type, research method, sample size, participants’ age, the type of knowledge assessed, outcome variables, effect sizes, and key findings concerning consumers’ knowledge (see Table A1). 

Additionally, the same two authors independently conducted a quality assessment of each included study. Due to the lack of standardized critical appraisal criteria for social science research, we developed the quality assessment instrument based on Petticrew and Robert’s [27] framework for appraising a survey and the Joanna Briggs Institute’s Critical Appraisal Checklist for Analytical Cross-Sectional Studies [28]. We used three categories to appraise the risk of bias: High (i.e., study reached “yes” scores of 49%); Moderate (i.e., study reached “yes” scores of 50% to 69%); and Low (i.e., study reached “yes” scores of more than 74%). We provide the quality assessment instrument and the risk of biases for each included study in Supplementary Material Table S2. 

### 2.5. Data Synthesis

To complete the systematic review, we qualitatively synthesized the descriptive statistics and interpretations of inferential statistics reporting consumers’ knowledge of functional foods and the factors that influence their knowledge. We identified the type of consumer knowledge that each study investigated and used the constant comparative method to categorize knowledge types into distinct groups [29]. We extracted the percentages reporting consumers’ knowledge levels to determine the range of knowledge levels pertaining to each knowledge category. In addition, we identified and combined each of the factors reported that were found to influence consumers’ knowledge. 

### 2.6. Data Analysis

We conducted a correlational meta-analysis and extracted correlation coefficient(s) (*r*) from each study to use as the estimated effect size. Eighteen studies (*n* = 18) provided sufficient quantitative data to be included in the meta-analysis. Of the 18 studies, five (*n* = 5) directly reported the correlation coefficient (*r*) [8,21,30,31,32]. Another nine studies (*n* = 9) reported regression coefficients that are appropriate for use in a meta-analysis, according to Peterson and Brown [5,22,33,34,35,36,37,38,39,40]. In addition, we calculated the correlation coefficients for two studies (*n* = 2) using the equations recommended by Borenstein et al. [41], which relied on means, standard deviations, and *t*-test results [20,42]. We also acquired correlation coefficients from two studies (*n* = 2) by connecting with the authors via email [17,43]. The authors of eight additional studies did not provide data for calculating effect sizes.

To complete the meta-analysis, we used R Studio software to conduct a random-effects model to analyze 18 studies (27 effect sizes) [44]. A random-effects model assumes all included studies are from different populations in which the average effect size varies randomly across studies [44,45]. Additionally, a random-effects model has unconditional inferences, allowing results obtained from the analysis to be generalized beyond the population of studies included in the meta-analysis [44]. We chose to use a random-effects model because the studies we included were conducted independently and their participants represented different populations. Therefore, through our study, we attempted to generalize results and conclusions. We also computed a funnel plot of effect sizes, in which standard error is plotted against the effect size measures, to visually examine the possibility of publication bias.

To interpret effect size homogeneity across studies, we relied on Cochran’s Q statistic and the I^2^ index [46]. Cochran’s Q statistic indicates the statistical significance of heterogeneity; however, when the number of included studies is small, its ability to detect true homogeneity is low [41,47]. Therefore, in addition to Cochran’s Q statistic, the I^2^ index was measured to report the extent of heterogeneity. The I^2^ index, which is not sensitive to the number of included studies, is reported as a percentage and represents the proportion of total variance across studies due to heterogeneity [46]. Generally, an I^2^ index of 75% or higher indicates high variation [46].

## 3. Results

### 3.1. Characteristics of Included Studies

The systematic review included 40 studies, 18 of which were included in the meta-analysis. Most of the reviewed studies were conducted in Europe (*n* = 23), six studies in Asia (*n* = 6), five studies in North America (*n* = 5), three studies in South America (*n* = 3), two studies in three different countries (i.e., Canada, U.S., France; *n* = 2), and one study in Africa (*n* = 1). All studies were conducted using survey methodology, including questionnaire surveys (*n* = 36) and survey-based economic evaluation techniques (i.e., experimental auctions (*n* = 2); choice experiments (*n* = 1)). One study used a mixed-method design (*n* = 1), part of which was also a questionnaire survey. The functional foods under investigation in the reviewed studies included functional meats, beverages, dairy products, and snacks (e.g., functional cookies, functional protein bars, functional cereal bars). 

### 3.2. Qualitative Systematic Review Findings

We identified three categories of consumer knowledge based on the focus of each reviewed study: knowledge of the concept of functional foods (i.e., knowledge of functional food definition, knowledge of functional food description), knowledge about nutrition associated with consuming functional foods (i.e., knowledge about nutrition, knowledge about diet-related issues, knowledge about health claims), and knowledge of specific functional food products (i.e., knowledge about specific functional foods, knowledge about functional ingredients, knowledge about functional foods brands). It is important to note that some studies measured consumers’ perceived knowledge, and some studies measured consumers’ actual knowledge. For example, Verbeke et al. (2009) measured consumers’ perceived knowledge of the concept of functional foods. They included the question, “As compared to other people of your age with a similar background, how do you estimate your knowledge about functional foods?” with response options ranging from ‘very low’ to ‘very high’. Sääksjärvi et al. (2009), on the other hand, measured consumers’ actual knowledge of the concept of functional foods and included the question “What are functional foods?” with ‘gene manipulated foods’, ‘food with added beneficial ingredients’, ‘food in capsules or pills’, ‘ecological food’, and ‘I don’t know’ as response options. Some study authors, however, did not provide information about how they measured knowledge, preventing us from determining whether it was actual or perceived. Still, based on what we do know and for the purpose of our study, consumer knowledge included actual and perceived knowledge. Moreover, the outcome variable in each study was consumer acceptance of functional foods, which was defined and described using different concepts. These included general acceptance, willingness to pay or purchase, intent to purchase, likelihood to purchase, frequency of consumption, and frequency of purchase.

#### 3.2.1. Consumers’ Knowledge of the Concept of Functional Foods

Consumers’ knowledge has been identified as an important predictor of their functional food acceptance. However, most studies indicated that participants had limited knowledge about or were not familiar with functional foods [48,49]. Five studies (*n* = 5) reported participants’ knowledge of the concept of functional foods. Specifically, the percentage of participants across studies knowledgeable about functional foods ranged from 21% to 52.3%. For example, Brečić et al. [17] found that only 21% of 424 Croatian participants believed they were ‘very well informed’ about functional foods, and Kolodinsky et al. [50] found that only 33.1% of 811 U.S., Canadian, and French participants had knowledge of functional foods. Similarly, 26.4% of 372 Chilean participants were knowledgeable about functional foods in Schnettler et al.’s study [51], and 40.6% of 251 Lebanese participants were knowledgeable about functional foods in Chammas et al.’s study [52]. Grochowska-Niedworok et al. [53] found that a higher percentage of 300 Polish participants (52.3%) were knowledgeable, and Stojanovic et al. [39] found more than half of Montenegrin participants (52% of 479) were moderately informed about functional foods.

Although most participants across studies were not knowledgeable about functional foods, they still showed interest in consuming functional foods [53]. Italian participants were more willing to accept functional foods if they were first exposed to the concept of functional foods [37]. In addition, providing appropriate health information about the health benefits of consuming functional foods could help consumers become more accepting [37]. For example, Dean et al. [34] found that consumers who were previously exposed to health information (i.e., how consuming functional foods could reduce disease risk) were more likely to accept functional foods.

#### 3.2.2. Consumers’ Knowledge about Nutrition of Functional Foods

In addition to general knowledge of functional foods, several studies reported consumers’ level of nutritional knowledge. For example, O’Connor and Venter [54] found that only 17.3% of 139 South African participants believed they had a high level of nutritional knowledge. In two other studies investigating consumers in the Republic of Macedonia [55] and Canada [56], participants’ nutritional knowledge was moderate. Moreover, Barreiro-Hurlé et al. [30] found that 45% of 300 Spanish participants were familiar with fat and cholesterol content and daily caloric recommendations. Furthermore, Yalçın et al. [57] found that 65.7% of 293 Turkish participants were familiar with dietary fiber and foods, and 59.1% were knowledgeable concerning the health effects of consuming dietary fiber.

#### 3.2.3. Consumers’ Knowledge of Specific Functional Foods

Five studies (*n* = 5) reported consumers’ level of knowledge regarding specific functional foods (e.g., iron-fortified soy sauce, functional eggs, functional meat, functional coffee). Generally, this type of knowledge was limited among global consumers. For example, Sun et al. [31] found that only 15% of 1090 Chinese participants in urban areas were familiar with iron-fortified soy sauce. In contrast, only 3% of Chinese participants in rural areas had heard about iron-fortified soy sauce. In addition, Hayat et al. [58] found that 24.3% of 262 Pakistani participants had heard about functional nutrient-enriched designer eggs, Sandmann et al. [8] found that 34% of 840 German participants were familiar with vitamin D fortified foods, and Corso et al. [21] found that only 3.7% of 270 Brazilian participants had heard of functional soluble coffee. Finally, a study conducted in four European countries—Belgium, Netherlands, Italy, and Germany—found that more than half of 2057 participants (54.9%) were unfamiliar with nitrite added to functional processed meats [59]. 

#### 3.2.4. Factors Influencing Consumers’ Knowledge of Functional Foods

Demographic characteristics (i.e., age, gender, educational level, marital status, nationality) influence consumers’ level of knowledge of functional foods. For example, Corso et al. [21] found that participants who were older and had higher education were more knowledgeable about the health benefits of consuming coffee enriched with antioxidants compared to participants who were younger and had less education. In addition, Cukelj et al. [7] found that female participants had higher nutritional knowledge than their male counterparts, and that female participants who had received a higher education had more nutritional knowledge when compared to females who had received less education. Although Cukelj et al. [7] found no association between participants’ knowledge and age, Chammas et al. [52] found that young people between the ages of 18 and 29, or single, had more knowledge of functional foods compared to people between the ages of 30 and 66 or married. Similarly, Hayat et al. [58] found that participants’ marital status significantly influenced their knowledge of specific functional foods, but their gender and educational level did not. Moreover, several studies found that consumers’ nationality influenced their knowledge. Specifically, Labrecque et al. [36] found that French participants had less knowledge about the term functional foods when compared to American and French-Canadian participants, and Kolodinsky et al. [50] found that American participants had more knowledge of functional foods when compared to French and Canadian participants. 

#### 3.2.5. Relationship between Consumers’ Knowledge and Their Acceptance of Functional Foods

Eighteen (*n* = 18) studies investigated the relationship between consumers’ level of knowledge and their acceptance of functional foods. Thirteen studies (*n* = 13) found positive correlations between participants’ knowledge (i.e., knowledge of functional foods, nutritional knowledge, knowledge of functional food brands) and their acceptance of functional foods, whereas two studies (*n* = 2) found significant negative correlations between participants’ health and/or nutritional knowledge and their functional foods purchasing frequency. In addition, three studies (*n* = 3) found that participants’ subjective nutritional knowledge was not significantly related to their acceptance of functional foods. Still, Sparke et al. [60] explained that even though a correlation did not exist between consumers’ knowledge of functional foods and their acceptance of such foods, knowledge is an important factor that could ultimately influence consumer acceptance. 

### 3.3. Meta-Analysis Results

**Figure 2:**
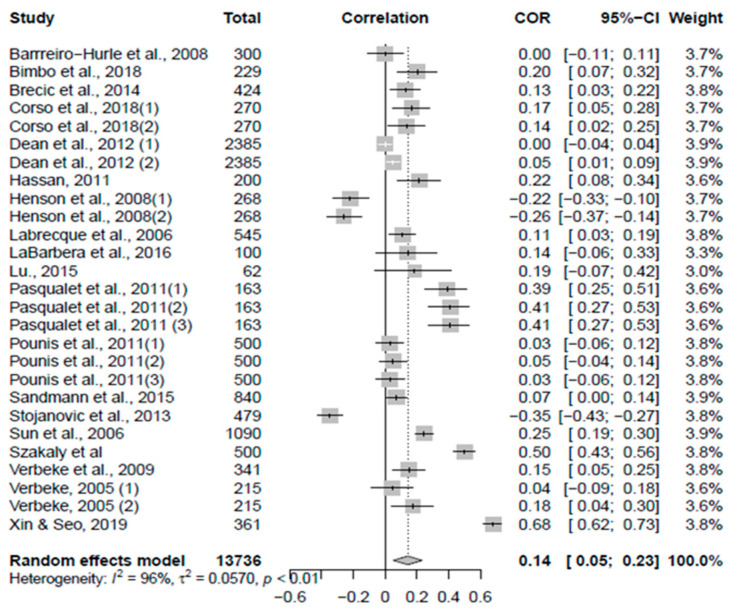
Forest plot of the 27 effect sizes (correlation coefficients (*r*)) with corresponding 95% confidence intervals [5,8,17,18,20,21,22,30,31,32,34,35,36,37,38,39,40,42,43].

**Figure 3:**
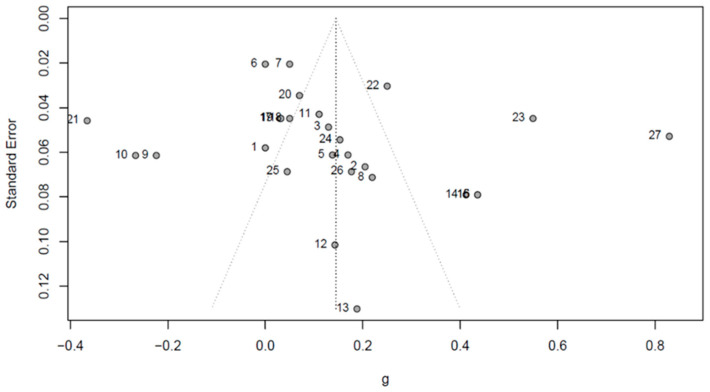
Funnel plot of the 27 effect sizes in which standard error is plotted against the effect size measures.

The 18 studies we included in the meta-analysis generated 27 effect sizes. These studies’ sample sizes ranged from 62 to 2385, and the total number of participants included in the meta-analysis was 13,736 (*N* = 13,736). We conducted a forest plot to view effect sizes across studies and determine their precision (see Figure 2). Results indicate that the pooled correlation effect size *r* between consumers’ level of knowledge and their acceptance of functional foods is 0.14 (*r* = 0.14, 95% CI = [0.05, 0.23], *z* = 3.05, *p* = 0.002), which represents a small effect, according to Funder and Ozer [61] and Gignac and Szodorai [62] who stated that 0.10 represents a small effect, 0.20 represents a medium effect, and 0.30 represents a large effect. Therefore, we found that a small, positive relationship exists between consumers’ knowledge and their acceptance of functional foods. In addition, results from Cochran’s Q test of effect size homogeneity (Q = 583.044, *df* = 26, *p* < 0.001) indicate that heterogeneity exists across studies. More specifically, results from the I^2^ index indicate that 95.5% [94.4%; 96.4%] of the variation across studies is due to heterogeneity. Results from the funnel plot show some asymmetry, which suggests publication bias may exist (see Figure 3).

## 4. Discussion

The first critical point of discussion for the study described herein is that promotion guided by the intent to build awareness is key to enhancing the conversation around and accepting functional foods. According to Wansink et al.’s [16] hierarchy of nutritional knowledge, consumers’ lack of knowledge could hinder their acceptance of functional foods. Urala and Lähteenmäki [15] found that consumers are more likely to accept functional foods if they understand the health benefits of consuming them. However, consumers need knowledge to evaluate and interpret information about health benefits adequately [63]. Therefore, helping consumers obtain knowledge is important because those who are more knowledgeable about functional foods and the health benefits associated with their consumption are significantly more likely to accept functional foods [5,17,34].

According to the studies included in this review, consumers’ knowledge of functional foods is limited. Most participants were not even aware of functional foods—they did not have adequate nutritional knowledge or understand the health benefits of consuming functional foods. Previous studies found consumers were unwilling to accept functional foods because they were uninformed about or found it difficult to understand nutritional information and health benefits [64,65]. In this regard, promoting the health benefits of, and nutritional information related to, functional foods is a key first step to increasing the public’s acceptance of such foods and, ultimately, their understanding of the relationship between diet and health. 

Previous studies indicate that increasing consumers’ nutritional knowledge could change their nutritional behaviors. As a result, this change could improve the population’s health status [66] and simultaneously lead to a wider acceptance of functional foods. Thus, effective scientific communication efforts are critical to disseminating information about nutrition and, ultimately, the adoption of healthy eating habits across the nation’s varying demographics. Agricultural and health communicators and educators play an instrumental role in increasing consumers’ access to information about the concept of functional foods, the health benefits associated with consuming functional foods, and the health risk associated with nutrition deficiencies [63,67], and often with increased access comes increased knowledge and a higher potential for behavior change. 

Because increased knowledge can lead to increased acceptance, we recommend the creation and implementation of a functional foods information campaign to increase consumers’ knowledge of functional foods and to educate consumers about the health benefits of consuming functional foods and the consequences of insufficient nutritional intake. This would likely be an effective approach because consumers are more willing to consume functional foods if they connect their knowledge to the health benefits of consuming them [16]. To reach broad audiences, including those more prone to accepting functional foods and those that would benefit most from consuming them, we recommend that social media, specifically Facebook, Twitter, and Instagram, be used as the primary platforms for dissemination. Because social media are an integral part of many people’s daily lives, they provide a low-cost way of reaching the masses with important health information [68,69]. As interactive spaces for scientific information dissemination, social media are effective modes for addressing public health nutrition issues and have the potential to influence healthy lifestyle behaviors [68].

Professionals in the functional foods industry should develop social media content that aims first to empathize with the group’s shared values to establish trust and then convey health information clearly and accurately [70,71]. The content should draw consumers’ attention to the connection between the health benefits of consuming functional foods and their personal health. Professionals should consider these specific strategies—use multimedia materials, include popular culture-inspired visual elements, and translate content into multiple languages—because these have been successful in other social media health promotion efforts [68]. Sufficient investment in educating consumers about comprehensive health information using social media could increase knowledge and lead to positives changes in nutritional behaviors [42,63,68].

Moreover, a cadre of school-based or secondary nutritional educators should provide curricula that include content about the health benefits of functional foods [72]. These curricula should help students better understand functional ingredients and products, provide adequate information about the consequences of insufficient nutritional intake, and increase their interest in consuming functional foods [73]. In addition, nutritional education programs should allow students to interact with industry scientists to learn how technology is used to develop functional foods. Better understanding will likely aid in counteracting consumer skepticism about such foods. Educators should also provide experiential learning opportunities for students to comprehend food science concepts [74], identify functional foods in grocery stores, and understand nutritional labels [75], ultimately helping students make healthy food choices.

The European Regulations on Food Information for Consumers recommend improving consumer knowledge of nutrition and labeling [76]. Providing explanatory health information (i.e., nutrient content, health benefits of essential nutrients) on food labels is the initial step in decreasing obesity [76,77]. Therefore, adults and educators should cooperate with dietitians and nutritionists to develop and deliver community-based nutrition education interventions [72]. These programs should include content about functional ingredients and products and explain the health benefits of consuming functional foods. For example, previous studies indicated that effective nutrition education inventions that include diet counseling, increasing access to health professionals, and distributing newsletters about healthy eating, can improve nutrition knowledge and result in positive behavior change across entire communities [72,78]. The benefits of school-based and adult consumer education programs should be evaluated holistically to document their impact [79].

A second important point of discussion is that the variation between effect sizes could be explained by the heterogeneity of the samples, the country of origin in which the studies were conducted, the types of functional foods used, and the unstandardized measurements of variables. Thus, we recommend that, when possible, future studies use nationally representative samples and standardized measurements to investigate the relationship between consumers’ knowledge and their acceptance of functional foods. In addition, using experimental research designs instead of survey methods can provide empirical causal evidence to explain the phenomenon using robust statistical methods. We also believe it would be valuable to include only studies in these meta-analyses that use specific populations, so we can begin to understand the differences in how these variables influence different consumer groups. Doing so would provide researchers with the data to segment audiences based on demographic and psychographic characteristics and, ultimately, develop audience-specific communications strategies, materials, and content. 

These future research efforts should rely on existing theoretical models related to health and behavior change to transfer research into practice more effectively. For instance, the health belief model, originally developed in the 1950s to explain people’s participation in disease detection and prevention programs, is now widely used to study people’s health [80]. Examples of constructs included in the model are modifying factors (e.g., ethnicity, education, knowledge), individual perceptions (e.g., perceived benefits, perceived barriers, self-efficacy), and actions (e.g., behaviors). With this model in mind, researchers could investigate how consumers’ knowledge of functional foods influences their perceived barriers to adoption. Rogers [81] diffusion of innovation theory would also enable researchers to investigate consumers’ acceptance of functional foods through a novel lens. The theory, which explains how a product diffuses through a social system, is based on five adopter categories: innovators, early adopters, early majority, late majority, and laggards. It would be interesting for researchers to investigate types and levels of knowledge that influence consumers’ rate of adoption (acceptance) and their likelihood to adopt (to accept) functional foods earlier than other persons. Results from basic theoretical research would inform practitioners about the types of knowledge consumers need to overcome perceived barriers to adopting functional foods and, ultimately, increase the rate at which they accept these foods. 

Because the current study has its limitations, we offer specific research recommendations to overcome them. Consumers’ acceptance of functional foods depends on a variety of interrelated factors, including consumers’ demographic characteristics (e.g., age, gender, educational level, marital status, nationality), affective domains (e.g., attitudes, perceptions), and situational domains (e.g., politics, economy) [82]. We focused only on consumers’ knowledge as a determinant of consumers’ acceptance. Other variables were beyond the scope of our research for the study described herein. Therefore, future systematic reviews and meta-analyses should focus on how other variables (e.g., attitudes, perceptions, motivations) influence consumers’ acceptance of functional foods so that the precise effect of these variables can be determined. 

As stated in a previous section, consumers’ demographic characteristics can influence their knowledge of functional foods. We extracted participants’ age from the included studies and reported this information in the summary table to provide context to our results (see Table A1). However, some of the included studies did not provide adequate information about their participants’ demographic characteristics, which hindered us from conducting further analyses using these characteristics. Therefore, we believe results from the meta-analysis reported herein provide information regarding the influence of consumers’ knowledge on their acceptance of functional foods that represents the general population. We recommend future meta-analyses include meta-regressions to investigate how consumers’ acceptance of functional foods could be explained by their demographic characteristics. 

In addition, the limited number of studies we included in the meta-analysis prevented us from conducting meta-regressions and moderator/mediator analyses. Therefore, future research should investigate potential moderators and mediators that might influence the relationship between consumers’ knowledge and their acceptance of functional foods. The more we know about this relationship, the more effectively we can communicate with specific audiences.

Findings from the systematic review and meta-analysis described herein provide novel and useful insight into the existing body of evidence regarding the effect of knowledge on consumers’ acceptance of functional foods. We independently analyzed the influence of knowledge types on consumer acceptance in the systematic review and collectively analyzed the influence of knowledge in the meta-analysis. As additional studies become published that investigate the relationship between consumers’ knowledge and their acceptance of functional foods, future meta-analyses should examine the effect of specific types of knowledge on consumer acceptance separately. However, for our meta-analysis, we chose to combine the three types of knowledge we identified in our literature review (i.e., knowledge of the concept of functional foods, knowledge about nutrition associated with consuming functional foods, knowledge of specific functional food products) seeking to generalize the meta-analysis results more broadly and because we were only able to include a small number of effect sizes. Qualitatively and quantitatively, we comprehensively synthesized the diverse findings from relevant studies. We added to the body of knowledge by providing practical recommendations for professionals in the functional foods industry and a clear trajectory for researchers to investigate varying dimensions of knowledge related to functional foods. 

## 5. Conclusions

A systematic review and meta-analysis are effective research methods to synthesize existing data and explore systematic evidence relating to a specific research topic [45]. To our best knowledge, this study is the first of its kind to investigate the relationship between consumers’ knowledge and their acceptance of functional foods using these methods. We identified a small, positive relationship between consumers’ level of knowledge and their acceptance of functional foods. The positive relationship we identified emphasizes the important role of consumers’ knowledge in their acceptance of functional foods. We also found that, for the most part, global consumers have low levels of knowledge related to functional foods, which is problematic due to its positive influence. Therefore, agricultural and health communicators and functional foods industry professionals, who play an instrumental role in increasing consumers’ access to information about functional foods, should strategically promote the health benefits of and nutritional information related to functional foods to increase public acceptance. We recommend this be done by implementing a functional foods information campaign using social media as primary platforms for dissemination, curricula for school-based or secondary nutritional educators, and community-based nutrition education interventions. These efforts to increase consumers’ knowledge would likely lead to increased acceptance and increased consumption, leading to the improved health and well-being of consumers across the globe. Because our study was limited to investigating consumers’ knowledge as a determinant of their acceptance, future systematic reviews and meta-analyses should examine the precise effect of demographic, affective, and situational factors on the same outcome through the lens of theoretical models related to health and behavior change. As a result, we will improve our ability to develop strategic messages for specific audiences and circumstances that can effectively improve their acceptance of functional foods.

## Data Availability

No new data were created or analyzed in this study. Data sharing is not applicable to this article.

## Supplementary Materials

The following supporting information can be downloaded at: https://www.mdpi.com/article/10.3390/foods11081135/s1. Table S1: Databases and search terms used to search articles. Table S2: Quality criteria of including studies for systematic review.

## Appendix A

**Table A1:** Characteristics of the Included Studies.

Study	Country	Functional Foods Type	Study Design	Knowledge Type	Outcome Variable	Correlation	Sample Size	Age of Participants	Risk of Bias	Main Findings about Knowledge
Arenna et al., 2019 [56]	Canada	Enhanced carnosine in pork	Surveyed-based choice experiment	Nutrition knowledge	Willingness to pay	Data was unusable	992	18+; Average age = 52.6	Low	Participants’ average nutrition knowledge score was 15.8 (ranging from 5–25); Participants’ level of nutrition knowledge is significantly, positively associated with their willingness to pay for functional foods.
Ares et al., 2008 [66]	Uruguay (South America)	16 functional foods concepts	Survey	Nutrition knowledge (nutrient content; antioxidants; connection between diet and diseases)	Willingness to try	Data was unusable	104	18–81; Average age = 34.3	Low	Nutrition knowledge significantly affected participants’ willingness to try functional foods; Nutrition knowledge significantly affected participants’ perceived healthiness of functional foods; Participants who had a low level of nutrition knowledge were not interested in consuming functional foods; Participants with a high level of nutrition knowledge were interested in healthy foods enriched with fiber or antioxidants.
Barreiro-Hurlé et al., 2008 [30]	Spain	Resveratrol-enriched red wine	Surveyed-based choice experiment	Nutrition knowledge	Willingness to pay	*r* = −0.01	300	Average age = 46.5	Low	45% of participants had nutrition knowledge regarding fat and cholesterol content and daily caloric recommendations; Participants who know the relationship between health and diet are more likely to buy functional wine.
Bimbo et al., 2018 [43]	Italian	Functional yogurts	Post-purchased survey	Knowledge about leading functional yogurt brands	The numbers of functional yogurt packages purchased	*r* = 0.2019	229	18–60	Low	Participants with more knowledge of leading functional yogurt brands purchased a higher number of functional yogurt packages; Regardless of participants’ level of knowledge of leading functional yogurt brands, those who did not like their own bodies were less likely to purchase functional yogurt packages.
Brečić et al., 2014 [17]	Croatia	The concept of functional foods	Self-administered survey	Knowledge of functional foods	Functional foods consumption frequency	*r* = 0.129	424	18+; Average age = 47.6	Low	6% of participants reported themselves as “fully informed” about functional foods; 21% reported themselves as “very well informed” about functional foods; A significant, positive relationship existed between participants’ knowledge of functional foods and their consumption of functional foods.
Chammas et al., 2019 [52]	Lebanon	Prebiotic yogurt; Protein bars; Protein shakes; Cereal bars	Survey	Knowledge of functional foods and functional ingredients	Functional foods acceptance	Data was unusable	251	34.5 ± 12.1	Low	40.6% of participants were knowledgeable about functional foods; 32% of participants were knowledgeable about functional ingredients; Participants between the ages of 18 and 29 had a higher knowledge level of functional foods; Single participants had a higher knowledge level of functional foods; Participants who went to the gym had a higher knowledge of functional foods.
Clark et al., 2019 [48]	England	Vitamin D fortified foods	Mixed methods (focus groups and survey)	Knowledge of vitamin D	Perceptions of fortified foods	Data was unusable	109	16+	Low	Participants had basic knowledge of vitamin D; Participants lacked knowledge about the health benefits of vitamin D sufficiency.
Corso et al., 2018 [21]	Brazil	Coffee enriched with antioxidants	Self-administered survey	Knowledge of functional foods	Functional foods acceptance	*r* = 0.168 (“if they taste good”); *r* = 0.137 (“if they taste worse than their conventional counterpart foods”) (p. 5)	270	Average age = 39.1	Low	Participants’ knowledge of functional foods was significantly, positively associated with their acceptance of functional coffee; 49.6% of participants knew the benefits of coffee ingestion; 60.7% of participants knew the benefits of antioxidants ingestion; 5.6% of participants knew the benefits of consuming soluble coffee.
Cukelj et al., 2016 [7]	Croatia	Flaxseed-enriched cookies	Online survey	Nutrition knowledge about lignans and omega-3 fatty acids (ingredients)	Purchase interests	Data was unusable	1035	15–65	Moderate	Female participants had a higher level of nutrition knowledge compared to male participants; Participants’ age was not associated with their knowledge level; Female participants’ educational level was significantly, positively associated with their level of nutrition knowledge; Participants with a higher level of nutrition knowledge consumed more functional cookies compared to those with a lower level of nutrition knowledge.
Dean et al., 2012 [34]	Finland; the UK; Germany; Italy	Bread, cake, and cereal-containing yogurt + benefit claim, risk reduction claim, and nutrition claim	Paper and pencil survey	Subjective knowledge	Likelihood to buy	*r* = 0.05 (nutrition claim); *r* = 0.04 (risk reduction claim)	2385	35–95; Average age = 52.1	Low	Participants’ subjective knowledge was a significant predictor of their likelihood to buy functional foods with a nutrition claim; Participants’ subjective knowledge did not increase their likelihood to buy functional foods with risk reduction claims.
Di Talia et al., 2018 [49]	Italy; Germany	The term functional foods	Survey	Knowledge of functional foods	Attitudes toward functional foods	Data was unusable	230	Data was not available	Moderate	Participants’ level of knowledge of functional foods was low; 68% of participants were informed consumers who had knowledge of functional foods.
Grochowska-Niedworok et al., 2017 [53]	Poland	The concept of functional foods	Survey	Knowledge of functional foods	Functional foods consumption	Data was unusable	300	Data was not available	Moderate	Participants’ level of knowledge about functional foods was low; 83.3% of participants did not know the amount of functional foods available on the market; 43.1% of healthy participants and 53.97% of participants with diseases had no knowledge regarding their consumption of functional foods.
Hasnah 2011 [35]	Malaysia	The concept of functional foods	Self-administered survey	Knowledge of functional foods	Functional foods consumption	*r* = 0.216	200	18–54	Low	Participants’ knowledge of functional foods positively influenced their functional foods consumption; Participants were knowledgeable about functional foods.
Hayat et al., 2010 [58]	Pakistan	Nutrient enriched designer eggs	Survey	Knowledge about designer eggs (type of functional food)	Perception and willingness to buy	Data was unusable	262	18+; Median age = 37	Moderate	14.2% of participants knew of nutrient-enriched designer eggs, and 85.7% did not; Male participants had slightly more knowledge than female participants; Participants’ marital status and occupation were significantly associated with their level of knowledge.
Henson et al., 2008 [22]	Canada	Tomato juice and a snack bar containing lycopene	Self-administered survey	Knowledge of medicine, nutrition, or health care	Purchase intention	*r* = −0.22 (tomato juice); *r* = −0.26 (snack bar)	268	18+	Low	Participants’ knowledge of medicine, nutrition, or health care was significant, and negatively associated with their intent to buy functional foods.
Herath et al., 2008 [83]	Canada	Food/beverage containing desirable nutritional qualities (fiber, antioxidants, essential fatty acids)	Survey	Knowledge about food-health linkages	Functional food receptiveness	Data was unusable	1753	18+	Low	Participants who had a higher level of knowledge of age-related diseases had greater receptivity toward functional foods.
Hung et al., 2016 [59]	Belgium; Netherlands; Italy; Germany	Meat products processed with natural compounds and a reduced level of nitrite	Survey	Objective knowledge about the purpose of adding nitrite to meat	Purchase intention	Data was unusable	2057	18–75; Average age = 45.5	Low	54.9% of participants had no knowledge about nitrite added to processed meats product.
Kolodinsky et al., 2008 [50]	Canada; United States; France	Eggs with omega-3; milk with calcium; orange juice with calcium	Self-administered survey	Knowledge of functional foods	Purchase intention	Data was unusable	811	22.4 ± 3.3	Low	33.1% of participants had good knowledge of functional foods; 28.1% had partial knowledge; 38.3% had no knowledge; Participants from the United States had greater knowledge about functional foods than French and Canadian participants.
Labrecque et al., 2006 [36]	Canada; United States; France	Milk with Omega-3; egg with Omega-3	Self-administered survey	Knowledge of the term ‘functional foods’	Functional food acceptance	*r* = 0.11	545	18–25	Low	56.9% of American participants, 45.8% of Canadian participants, and 10.6% of French participants knew the term functional foods; Participants’ level of functional foods knowledge was significantly, positively associated with their acceptance of functional foods.
La Barbera et al., 2016 [5]	Italian	Crushed tomatoes enriched with lycopene	Surveyed-based experimental auction	Subjective knowledge about lycopene	Willingness to pay	*r* = 0.1423	100	Average age = 23.06	Low	Participants’ level of knowledge about lycopene was significantly, positively associated with their willingness to pay a higher premium price for functional foods.
Lu., 2015 [20]	Canada	30 hypothetical functional foods	Surveyed-based experimental auction	Nutrition knowledge	Purchase intention	*r* = 0.1862	62	18–55	Low	Participants’ level of nutrition knowledge was significantly, positively associated with their intent to purchase functional foods; Participants’ level of nutrition knowledge significantly moderated the relationship between their perceived carrier-ingredient fit of a functional food and their purchase intention.
Nguyen, 2020 [84]	Vietnam	The term functional foods	Survey	Knowledge of functional foods	Functional food acceptance	Data was unusable	260	20+	Low	Participants’ level of functional foods knowledge was significant, and positively associated with their acceptance of functional foods.
O’Connor & Venter, 2012 [54]	South Africa	Ten bioactive food ingredients (functional ingredients)	Survey	Health and wellness knowledge; Nutrition knowledge	Perceived interest	Data was unusable	139	25–65	Low	22.3% of participants perceived their level of health and wellness knowledge to be well informed; 66.9% of participants perceived their level of health and wellness knowledge to be moderately informed; 17.3% of participants perceived their level of nutrition knowledge to be well informed; 65.5% of participants perceived their level of nutrition knowledge to be moderately informed; Participants who had higher knowledge of Omega-3 fatty acids, probiotics, and soy protein tended to adopt functional foods.
Di Pasquale et al., 2011 [37]	Italy	Milk, butter, and yogurt fortified with conjugated linoleic acid	Self-administered survey	Knowledge of the relationship between diet and health; Knowledge of functional foods	Willingness to pay	*r* = 0.39 (milk); *r* = 0.41; (yogurt); *r* = 0.41 (butter)	163	20–80; Average age = 43	Low	Participants’ knowledge of functional foods significantly influenced their willingness to pay for functional foods; 29% of participants had no knowledge of functional foods or the relationship between diet and health; 29% of participants knew the major functional foods product categories and some knowledge of the relationship between diet and health; 28% of participants had some knowledge of functional foods that was greatly influenced by advertising and no knowledge of the relationship between diet and health; 14% of participants knew of (were familiar with) functional foods and knew (good awareness) of the relationship between diet and health.
Pounis et al., 2011 [38]	Greece	Iron-fortified foods	Survey	Overall nutrition knowledge; general nutrition knowledge; iron nutrition knowledge	Iron fortified foods perception and consumption	*r* = 0.03 (overall nutrition); *r* = 0.05 (general nutrition); *r* = 0.032 (iron deficiency)	500	30 ± 12	Low	Increasing participants’ overall nutrition knowledge improved their perception of iron-fortified foods; Participants’ overall nutrition knowledge, general nutrition knowledge, and iron nutrition knowledge were significantly, positively associated with their consumption of iron-fortified foods.
Sääksjärvi et al., 2009 [85]	Finland	The term functional foods	Survey	Knowledge of functional foods	Purchase behavior	Data was unusable	409	18+	Low	Participants’ attitudes toward health mediated the effect between their knowledge of functional foods and purchase behavior; Female participants had more knowledge of functional foods than male participants; Participants aged 55 to 65 had the most knowledge of functional foods; Participants’ income was significantly, positively associated with their knowledge of functional foods; University-educated participants had more knowledge of functional foods than high school-educated participants.
Sandmann et al., 2015 [8]	Germany	Vitamin D-fortified foods (i.e., juice, cereals, butter, milk, yogurt)	Online survey	General knowledge of vitamin D	Acceptance of vitamin D-fortified foods	*r* = 0.07	840	19+	Low	Participants’ general knowledge about vitamin D was not significantly related to their acceptance of vitamin D-fortified foods; Most participants lacked general knowledge about vitamin D; 22% of participants reported that their vitamin D-related knowledge was good.
Schnettler et al., 2015 [86]	Chile	Functional foods with 18 health benefits	Survey	Knowledge of functional food	Willingness to buy	Data was unusable	400	<35 (34.5%); 35–54 (42.0%); 55 + (23.5%)	Low	Participants’ level of functional foods knowledge positively influenced their willingness to buy.
Schnettler et al., 2016 [51]	Chile	Data was not available	Survey	Knowledge of functional food	Attitude	Data was unusable	372	Average age = 20.4	Low	Most participants (83.6%) had no prior knowledge of functional foods.
Sparke et al., 2009 [60]	Germany; Poland; Spain; United Kingdom	Orange juice enriched with functional ingredients recombined with different health claims	Survey	Knowledge of functional food	Functional food purchase frequency	Data was unusable	590	Data was not available	Low	There was no correlation between participants’ knowledge of functional foods and their purchase frequency.
Spiroski et al., 2013 [55]	Republic of Macedonia	Data was not available	Survey	Nutrition knowledge	Attitude	Data was unusable	518	18+	Low	Participants’ nutrition knowledge was at a moderate level.
Stojanovic et al., 2013 [39]	Montenegro	Products with health claims (e.g., the benefits of high calcium)	Self-administered survey	Knowledge of foods with health claims	Consumption frequency	*r* = −0.35	479	18+	Low	52% of participants were moderately informed about foods with health claims; 1.9% of participants were fully informed; 8.6% of participants were not informed at all; Participants’ level of knowledge of foods with health claims is a predictor of their functional food consumption.
Sun et al., 2006 [31]	China	Iron-fortified soy sauce	Survey	Knowledge of iron-fortified soy sauce	Purchase intention	*r* = 0.245	1090	Average age = 37.33	Low	Participants had limited knowledge of iron-fortified soy sauce; 3% of participants from rural areas and 15% of participants from urban areas had heard of iron-fortified soy sauce; Participants’ knowledge of iron-fortified soy sauce was significant, and positively associated with their intention to purchase.
Szakály et al., 2019 [40]	Hungary	Dairy-based probiotic products (e.g., yogurt, cheese, muesli)	Self-administered survey	Knowledge of functional foods	Willingness to pay	*r* = 0.5	500	18–69	Low	Participants’ subjective knowledge of functional foods was significant, and positively associated with their purchase patterns of functional dairy products.
Verbeke et al., 2009 [42]	Belgium	Calcium-enriched fruit juice; Omega-3 enriched spread; fiber-enriched cereal	Self-administered survey	Knowledge of functional foods	Intention to buy product	*r* = 0.15	341	Average age = 37.4	Low	There was no significant relationship between participants’ knowledge of functional foods and their acceptance.
Verbeke, 2005 [18]	Belgium	The concept of functional foods	Self-administered survey	Knowledge of functional foods	Functional foods acceptance	Data was unusable	215	Average age = 39.1	Low	There was no significant relationship between participants’ knowledge of functional foods and their acceptance.
Verneau et al., 2019 [87]	Italy	Canned crushed tomatoes enriched with lycopene	Experimental auction	Knowledge about lycopene (ingredients)	Willingness to pay	Data was unusable	100	Average age = 23.88	Low	Participants with low knowledge of lycopene increased their willingness to pay after receiving information about the product.
Wansink et al., 2005 [16]	Canada; United States	The term functional foods	Mail survey	Knowledge of soy (attribute-related knowledge; consumption consequence-related knowledge)	Functional foods consumption	Data was unusable	606	Data was not available	Low	74.4% of participants had attribute-related knowledge, consumption consequence-related knowledge, or both; participants with attribute-related knowledge and consequence-related knowledge were more likely to consume soy products.
Xin & Seo, 2019 [32]	China	Korean functional foods (e.g., red ginseng, ginseng, vitamin, tonic, calcium, fish oil)	Online survey	Subjective knowledge of Korean functional foods	Purchase intention	*r* = 0.68	361	20–60	Low	Participants’ subjective knowledge about Korean functional foods was significant, and positively associated with their purchase intention.
Yalçın et al., 2020 [57]	Turkey	Foods with dietary fibers	Survey	Knowledge of dietary fibers and foods; knowledge of dietary fibers and health effects	Attitude	Data was unusable	293	18–71; Average age = 34.8	Low	65.7% of participants had a high level of knowledge about dietary fibers and foods; 59.1% of participants had a high level of knowledge about dietary fibers and health effects.
