# Supplementary material for: Investigating the Effect of Consumers’ Knowledge on Their Acceptance of Functional Foods: A Systematic Review and Meta-Analysis
Source: Foods. 2022 Apr 14;11(8):1135. doi: 10.3390/foods11081135 (PMC9028956; doi:10.3390/foods11081135)
Supplement: Supplementary file 1 [file foods-11-01135-s001.zip › foods-1668910-supplementary.pdf]

Supplementary Material 1

TableS1 Databases and search terms used to search articles

Database (Search date)	Search terms	No. of articles
CAB Abstracts (Ovid) (July 6, 2021)	((functional food* or functional product* or enriched food* or enriched product* or fortified food* or fortified product* or enhanced food* or enhanced product*).mp AND (accept* or behavior* or attitude* or perception* or pay or buy or purchase* or preference* or choice* or response*or reaction* or aware* or believe* or belief).mp AND (knowledge).mp.	154
FSTA (15 July 2021)	**Subject Heading search**: 88<br><br>(DE "FUNCTIONAL FOODS" OR DE "FUNCTIONAL BEVERAGES" OR DE "FORTIFIED BEVERAGES" OR DE "FORTIFIED FOODS") AND (DE "CONSUMER ACCEPTABILITY" OR DE "CONSUMER ACCEPTANCE" OR DE "CONSUMER ATTITUDES" OR DE "CONSUMER AWARENESS" OR DE "CONSUMER BEHAVIOUR" OR DE "PURCHASING BEHAVIOUR" OR DE "CONSUMER CHOICE" OR DE "CONSUMER EXPECTATIONS" OR DE "CONSUMER LIKING" OR DE "CONSUMER OPINIONS" OR DE "CONSUMER PERCEPTION" OR DE "CONSUMER PREFERENCE") AND ((DE "NUTRITIONAL LABELLING") OR knowledge)<br><br>**Title/Abstract Search:** 118<br><br>((TI ("FUNCTIONAL FOOD*" OR "FUNCTIONAL BEVERAGE*" OR “FUNCTIONAL PRODUCT*” OR "FORTIFIED FOOD*" OR "FORTIFIED BEVERAGE*" OR “FORTIFIED PRODUCT*” OR “ENRICHED FOOD*” OR “ENRICHED PRODUCT*” OR “ENHANCED FOOD*” OR “ENHANCED PRODUCT*”)) OR (AB ("FUNCTIONAL FOOD*" OR "FUNCTIONAL BEVERAGE*" OR “FUNCTIONAL PRODUCT*” OR "FORTIFIED FOOD*" OR "FORTIFIED BEVERAGE*" OR “FORTIFIED PRODUCT*” OR “ENRICHED FOOD*” OR “ENRICHED PRODUCT*” OR “ENHANCED FOOD*” OR “ENHANCED PRODUCT*”))) AND ((TI (ACCEPT* OR BEHAVIOR* OR BEHAVIOUR OR ATTITUDE* OR PERCEPTION* OR PAY OR BUY OR PURCHASE* OR PREFERENCE* OR CHOICE* OR RESPONSE*OR REACTION* OR AWARE* OR BELIEVE* OR BELIEF)) OR (AB (ACCEPT* OR BEHAVIOR* OR BEHAVIOUR OR ATTITUDE* OR PERCEPTION* OR PAY OR BUY OR PURCHASE* OR PREFERENCE* OR CHOICE* OR RESPONSE*OR REACTION* OR AWARE* OR BELIEVE* OR BELIEF))) AND ((TI ("NUTRITION* LABEL*" OR	206

	KNOWLEDGE)) OR (AB ("NUTRITION* LABEL*" OR KNOWLEDGE)))	
Web of Science (core collection) (22 July 2021)	("FUNCTIONAL FOOD*" OR "FUNCTIONAL BEVERAGE*" OR “FUNCTIONAL PRODUCT*” OR "FORTIFIED FOOD*" OR "FORTIFIED BEVERAGE*" OR “FORTIFIED PRODUCT*” OR “ENRICHED FOOD*” OR “ENRICHED PRODUCT*” OR “ENHANCED FOOD*” OR “ENHANCED PRODUCT*”) AND (ACCEPT* OR BEHAVIOR* OR BEHAVIOUR OR ATTITUDE* OR PERCEPTION* OR PAY OR BUY OR PURCHASE* OR PREFERENCE* OR CHOICE* OR RESPONSE*OR REACTION* OR AWARE* OR BELIEVE* OR BELIEF) AND (KNOWLEDGE)	690

Supplementary Material 2

Table S2 Quality criteria of including studies for systematic review

**Authors**	**Q1**	**Q2**	**Q3**	**Q4**	**Q5***	**Q6**	**Q7**	**Q8**	**Percentage of ‘Yes’**	**Risk of bias**
Arenna et al., 2019	Y	Y	Y	Y	N	Y	Y	Y	100%	Low
Ares et al., 2008	Y	N	y	Y	N	y	Y	Y	87.50%	Low
Barreiro-Hurlé et al., 2008	Y	Y	Y	Y	N	Y	Y	Y	100%	Low
Bimbo et al., 2018	Y	Y	Y	Y	N	Y	Y	Y	100%	Low
Brečić et al., 2014	Y	Y	Y	Y	N	Y	Y	Y	100%	Low
Chammas et al., 2019	Y	Y	Y	Y	Y	Y	Y	Y	87.50%	Low
Clark et al., 2019	U	Y	Y	Y	Y	Y	Y	Y	75%	Low
Corso et al., 2018	Y	Y	Y	Y	Y	Y	Y	Y	87.50%	Low
Cukelj et al., 2016	N	U	Y	Y	U	Y	Y	Y	62.50%	Moderate
Dean et al., 2012	U	Y	Y	Y	U	Y	Y	Y	87.50%	Low
Di Talia et al., 2018	U	U	Y	Y	U	Y	Y	Y	62.50%	Moderate
Grochowska-Niedworok et al., 2017	N	N	Y	U	U	Y	Y	Y	50%	Moderate
Hasnah 2011	N	Y	Y	Y	Y	Y	Y	Y	87.50%	Low
Hayat et al., 2010	U	N	Y	U	U	Y	Y	Y	50%	Moderate
Henson et al., 2008	U	Y	Y	Y	U	Y	Y	Y	75%	low
Herath et al., 2008	Y	Y	Y	Y	N	Y	Y	Y	100%	Low
Hung et al., 2016	Y	Y	Y	Y	N	Y	Y	Y	100%	Low
Kolodinsky et al., 2008	N	Y	Y	Y	Y	Y	Y	Y	75%	Low
Labrecque et al., 2006	N	Y	Y	Y	Y	Y	Y	Y	75%	Low
La Barbera et al., 2016	N	Y	Y	Y	Y	Y	Y	Y	87.50%	Low
Lu., 2015	N	Y	Y	Y	Y	Y	Y	Y	75%	Low
Nguyen, 2020	U	Y	Y	Y	U	Y	Y	Y	87.50%	Low
O'Connor & Venter, 2012	N	Y	Y	Y	Y	Y	Y	Y	75%	Low
Di Pasquale et al., 2011	N	Y	Y	Y	Y	Y	Y	Y	75%	Low

Pounis at al., 2011	N	Y	Y	Y	Y	Y	Y	Y	75%	Low
Sääksjärvi et al., 2009	U	Y	Y	Y	U	Y	Y	Y	87.50%	Low
Sandmann et al., 2015	Y	Y	Y	Y	N	Y	Y	Y	100%	Low
Schnettler et al., 2015	Y	Y	Y	Y	N	Y	Y	Y	100%	Low
Schnettler et al., 2016	N	Y	Y	Y	Y	Y	Y	Y	75%	Low
Sparke et al., 2009	N	Y	Y	Y	Y	Y	Y	Y	75%	Low
Spiroski et al., 2013	Y	Y	Y	Y	N	Y	Y	Y	100%	Low
Stojanovic et al., 2013	Y	Y	Y	Y	N	Y	Y	Y	100%	Low
Sun et al., 2006	N	Y	Y	Y	Y	Y	Y	Y	75%	Low
Szakály et al., 2019	Y	Y	Y	Y	N	Y	Y	Y	100%	Low
Verbeke et al., 2009	Y	Y	Y	Y	N	Y	Y	Y	100%	Low
Verbeke, 2005	N	Y	Y	Y	Y	Y	Y	Y	75%	Low
Verneau et al., 2019	N	Y	Y	Y	Y	Y	Y	Y	87.50%	Low
Wansink et al., 2005	Y	Y	Y	Y	N	Y	Y	Y	100%	Low
Xin & Seo., 2019	N	Y	Y	Y	Y	Y	Y	Y	87.50%	Low
Yalçın et al., 2020	N	Y	Y	Y	Y	Y	Y	Y	87.50%	Low

Note.
Q1: Is the sample surveyed representative?
Q2: Were the study sample and the setting described in detail?
Q3: Was the sample size large enough?
Q4: Was the measurement valid and reliable?
Q5: Is the survey method likely to have introduced significant bias? (e.g., sampling method could include random versus non-probability sampling).
Q6: Is there an adequate description of the data? (Including tables and summary statistics describing the sample, and adequate information on analysis results)
Q7: Were the outcomes measured in a valid and reliable way?
Q8: Are the statistical analyses appropriate?
* Recoded
Y = Yes; N = No; U = Unclear; NA = Not appliable
